# Anastasis: cell recovery mechanisms and potential role in cancer

**DOI:** 10.1186/s12964-022-00880-w

**Published:** 2022-06-03

**Authors:** Rebar N. Mohammed, Mohsen Khosravi, Heshu Sulaiman Rahman, Ali Adili, Navid Kamali, Pavel Petrovich Soloshenkov, Lakshmi Thangavelu, Hossein Saeedi, Navid Shomali, Rozita Tamjidifar, Alireza Isazadeh, Ramin Aslaminabad, Morteza Akbari

**Affiliations:** 1 Medical Laboratory Analysis Department, College of Health Sciences, Cihlan University of Sulaimaniya, Kurdistan Region, Sulaimaniya, Iraq; 2 College of Veterinary Medicine, University of Sulaimani, Sulaimaniyah, Iraq; 3 Department of Psychiatry and Clinical Psychology, Zahedan University of Medical Sciences, Zahedan, Iran; 4 Immunology Research Center, Tabriz University of Medical Sciences, Tabriz, Iran; 5 Department of Physiology, College of Medicine, University of Sulaimani, Sulaimaniyah, Iraq; 6 Department of Medical Laboratory Sciences, Komar University of Science and Technology, Sarchinar District, Sulaimaniyah, Iraq; 7 Department of Oncology, Tabriz University of Medical Sciences, Tabriz, Iran; 8 I. M. Sechenov First Moscow State Medical University, Ministry of Health of the Russian Federation (Sechenov University), Moscow, Russia; 9 Department of Pharmacology, Saveetha Dental College, Saveetha Institute of Medical and Technical Science, Saveetha University, Chennai, India

**Keywords:** Apoptosis, Anastasis, Chemotherapy, Drug resistance

## Abstract

Balanced cell death and survival are among the most important cell development and homeostasis pathways that can play a critical role in the onset or progress of malignancy steps. Anastasis is a natural cell recovery pathway that rescues cells after removing the apoptosis-inducing agent or brink of death. The cells recuperate and recover to an active and stable state. So far, minimal knowledge is available about the molecular mechanisms of anastasis. Still, several involved pathways have been explained: recovery through mitochondrial outer membrane permeabilization, caspase cascade arrest, repairing DNA damage, apoptotic bodies formation, and phosphatidylserine. Anastasis can facilitate the survival of damaged or tumor cells, promote malignancy, and increase drug resistance and metastasis. Here, we noted recently known mechanisms of the anastasis process and underlying molecular mechanisms. Additionally, we summarize the consequences of anastatic mechanisms in the initiation and progress of malignancy, cancer cell metastasis, and drug resistance.

**Video Abstract**

**Supplementary Information:**

The online version contains supplementary material available at 10.1186/s12964-022-00880-w.

## Background

Cancer occurs as the defect or abnormal regulation of the cellular pathways that involve cell growth, differentiation, and death. Identification and recognition of these cellular pathways have provided numerous methods for cancer therapy [1]. Current therapeutic approaches to cancer are surgery, radiotherapy, and chemotherapy drugs which commonly are based on induction of programmed cell death (apoptosis) and cell cycle arrest. However, epigenetic modifications, genetic background, and environmental factors can alter tumorigenesis pathways and cancer cells' response to therapeutic agents [2, 3].

Previous studies have reported the existence of numerous different signaling pathways that promote programmed cell death (apoptosis). The caspases are the most critical executors of apoptosis; however, other programmed and regulated signaling pathways are involved in this process [4–6]. The activated caspases cause cell death by cleaving several structural proteins. The essential cellular components and organelles are destroyed during apoptosis, indicating that apoptosis is an irreversible process [7]. Numerous studies have provided evidence indicating that apoptotic cells can be reverted from their destination. In this pathway, anastasis, apoptotic cells can recover to a stable condition after eliminating apoptosis-inducing drugs [8, 9]. A study by Tang et al. indicated that survival of breast cancer cells is possible after induction of apoptosis by ethanol; After-treatment of cancer cells by ethanol, some signs of apoptosis initiation were observed in treated cells, but apoptosis was not completed, and cells viability were restored [10]. Ichim et al. reported the cell restoration after induction of apoptosis by sublethal doses of various chemical compounds [11]. A similar study by Sun et al. suggested that investigation of molecular mechanisms of anastasis in normal cells can clarify specific molecular aberrations that may promote carcinomatous transformation and provide a novel potential approach for managing patients with cancer [12].

In the present review, we provide and summarize recently reported pieces of evidence that support the anastasis process and underlying probable molecular mechanisms. In addition, we noted the cancer cell metastasis and drug resistance as consequences of the anastatic process.

## Programmed cell death by apoptosis and necroptosis

Apoptosis is characterized by numerous morphological (cell shrinkage, membrane blebbing, chromatin condensation, and nuclear fragmentation) and molecular alterations [13]. Apoptosis is initiated through the intrinsic or extrinsic pathway, in which the caspase family is the most crucial component in this process [14]. In the intrinsic or mitochondrial apoptosis pathway, apoptotic cell death starts in the presence of intracellular stresses (endoplasmic reticulum stress, DNA damage, and growth factor deprivation). In the extrinsic pathway, apoptotic cell death is initiated in the presence of extracellular signals created by the ligation of death receptors to the plasma membrane [9]. In both intrinsic and extrinsic pathways, the caspase cascades are activated, cleave substrates of several proteins, and lead to quick cell death [15].

Mitochondria play is a critical role in apoptosis signaling, and both extrinsic and intrinsic pathways are dependent on mitochondrial outer membrane permeabilization (MOMP) [16]. Cellular depletion of mitochondria can severely affect apoptosis dynamics through mitophagy induction [17]. If MOMP occurs, cytochrome c is released into the cytosol and binds to the apoptotic protease activating factor 1 (APAF1). This allows caspase-9 to wrap to APAF-1 and form the apoptosome (a cytoplasmic protein complex). In addition, caspase-9 activates several executioner caspases (caspases-3, caspase-6, and caspase-7) through proteolytic cleavage. Finally, these executioner caspases lead to the completion of cell death [18, 19]. Moreover, the B-cell lymphoma 2 (Bcl-2) family members (three subfamilies that induce or inhibit apoptosis) play a pivotal role in the precise control of MOMP (14). The pro-apoptotic subfamily members (BAK, BAX, and BOK) are involved in mitochondrial outer membrane permeabilization and targeting. Moreover, the anti-apoptotic subfamily members (Bcl-2, Bcl-XL, and Mcl-1) prevent MOMP [20, 21].

Necroptosis is a programmed necrotic or inflammatory cell death that occurs in a caspase-independent manner and is mainly regulated by mixed lineage kinase domain-like (MLKL) and receptor-interacting proteins (RIPs). Necroptosis is an alternative mode of apoptosis that overcome resistance to apoptosis and may amplify and promote antitumor immunity in cancer treatment [22]. Evidence suggested that dysregulated necroptosis is associated with several pathological conditions such as inflammatory diseases, neurodegenerative diseases, and cancer [23, 24].

## Cell death recovery by anastasis

Previous studies suggested that several cells can reversely survive biochemical hallmarks of necroptosis and apoptosis [25, 26]. At the first time, Tang et al. reported that ethanol treatment could cause HeLa cervical cancer cells to survive after induction of apoptosis. However, critical signs of apoptosis were observed (DNA breaks, MOMP, and caspase-3 activations) in the cancer cells after ethanol treatment. Still, apoptosis was not completed in this case, and the viability and morphology of the cancer cells were restored [10]. Tang et al. introduced the term "anastasis" (rising to life) as cell survival after initiation of apoptosis [8]. Other studies reported further information on anastasis and hypotheses about underlying mechanisms of function, and novel approaches were developed to detect anastasis [27, 28]. Anastasis is commonly used for cell survival after initiating apoptosis in early and late stages. More precisely, anastasis is the survival and recovery of cells after induction of apoptosis [29]. However, there is minimal information on the functional mechanisms of cell viability and healing in anastasis. Therefore, further studies are essential to understand this process more accurately.

### Recovery through mitochondrial outer membrane permeabilization

Evidence suggested that MOMP is an apoptosis initiation point, and cell survival is impossible after this event. This process is responsible for to defect of mitochondria for energy generation, which causes to release of cytochrome c to cytosol, activation of the caspases, and DNA damage [30, 31]. However, it has been confirmed by previous studies that despite such destructive events, the cell can recover and restore normal function after MOMP [30, 32]. In this regard, the outer membrane in some mitochondria maintains integrity during permeabilization in apoptosis, which occur only upon initiation of apoptosis by various stimuli such as FAS-ligand (FASLG) [33], tumor necrosis factor-related apoptosis-inducing ligand (TRAIL) [34], tumor necrosis factor-alpha (TNFα) [35], bile acid [36], apoptosis inducer ABT-737, and proapoptotic BH3-mimetic, and proteasome inhibitor MG132 [37], staurosporine and U.V. radiation [38], etoposide and paclitaxel [39], and overexpression of Bcl-2 Homology 3 (BH3) interacting domain death agonist (BID) [40].

So far, the main mechanisms of mitochondria outer membrane integrity are not known; however, previous studies suggested that upregulation of antiapoptotic BCL2 family proteins plays a protective role in mitochondria integrity [41]. With MOMP, several mitochondria can maintain their integrity and produce the required energy for the cell [42]. At the late anastasis, the fragments of mitochondria are fused and recover intact morphology. However, the cells must deal with outcomes of MOMP (eliminate damaged mitochondria, remove proapoptotic factors, and inhibit cytosolic cytochrome c) for a successful anastasis [29].

Likely, anastatic cells eliminate cytosolic cytochrome c and damaged mitochondria through autophagy. The autophagy proteins (ATG12 and SQSTM1) are involved in the intensity of mitochondrial homeostasis and mitophagy. In the absence of caspase cascade, upregulation of the ATG12 is engaged in the rapid elimination of the cytosolic cytochrome c [43]. In addition, high expression of some heat shock proteins (HSPs) has been reported in murine hepatocytes during anastasis. Heat shock proteins (such as HSP90AA1, HSPB1, and HSPA1A) are chaperones produced by cells to respond to oxidative stress and detrimental thermal [44]. Both HSP27 and HSP70 chaperones prevent the cytochrome c secretion by mitochondria [45]. These proteins bind to hydrophobic residues created due to stress, prevent aggregation of partially denatured proteins, and promote refold of these proteins [46]. High Hsp70 and Hsp90 are common characteristics of thermotolerant cells that indicate an essential role for HSPs in inhibiting apoptotic stimuli [47, 48].

In addition, high expression of glyceraldehyde-3-phosphate dehydrogenase (GAPDH) protein in HeLa cervical cancer cells can recover mitochondrial outer membrane, promote cell division and proliferation after induction of apoptosis in the absence of caspase, and eliminate MOMP during anastasis [49]. The anti-apoptotic activity of GAPDH is observed only in the absence of activated caspases and plays an essential role in removing MOMP during anastasis [50]. This factor also plays a critical role in determining cancer cell fate. Furthermore, GAPDH is a cell death regulator that participates in tumor progression [51, 52].

These factors potentially play a role in the anastasis pathway, including the degradation of cytosolic cytochrome c, the elimination of damaged mitochondria, and the prevention of complete mitochondrial apoptosis.

### Recovery through caspase cascade arrest

The effector caspases cascade, apoptosis initiation pathways, is central in apoptotic cell death. Previously, the effector caspases activation was believed to be irreversible because effector caspases maintain apoptosis through positive feedback [53]. However, recent evidence demonstrated that anastasis could initiate even after activating the effector caspase cascade. For example, previous studies indicated that the caspase-3 was activated after apoptosis induction by ethanol treatment in vitro and in vivo. However, the cells recovered normal cellular function and morphology after removing ethanol treatment [8]. Evidence suggested that this recovery of apoptotic can be due to the limited role of caspases-9, caspases-7, and caspases-3 by MOMP [54]. In this case, the cell should remove the activated caspases in the cytosol. The caspases' elimination mechanisms are unknown; however, a group of HSPs is remarkable. The activation of procaspase-3 is inhibited by HSP27 protein. Moreover, activation of caspase-9 and apoptosome formation is suppressed by HSP27, HSP70, and HSP90 proteins [19, 55].

High expression of the double mouse minute 2 homolog (MDM2) gene, a ubiquitin ligase that directs p53 to degradation of the proteasome, is one of the main characteristics of anastatic cells upregulation [56], causing downregulation of the proapoptotic BAX gene. In addition, MDM2 causes upregulation of the antiapoptotic X-linked inhibitor of apoptosis (XIAP) gene, which leads to suppression of the effector and initiator caspases [57]. However, further studies are required to achieve more detail to specify the involved factors in anastasis characterized processes and non-lethal apoptotic functions.

### Recovery through repairing DNA damage

A wide range of chromosomal aberrations and DNA breakage is found in most anastatic cells, increasing cancer risk. Several anastatic factors (MDM2, CAD, and ICAD) play a vital role in preventing DNA damage [58, 59]. The expression level of ubiquitin ligase MDM2 is enhanced in anastatic cells, which degrades p53 protein and suppresses p53 associated mitochondrial apoptosis and DNA damage [60]. Moreover, expression of both DNA repair protein Poly (ADP-ribose) polymerase (PARP) and inhibitor of caspase-3– activated DNase (ICAD) increased in anastatic cells, which returns to baseline after remove of apoptosis induced factor [61].

Previous studies reported that expression of histone coding genes (Hist1h2ah, Hist1h2ad, and Hist1h2ai) decreases after removal of the proapoptotic agent, which can be associated with cellular response to DNA damage during anastasis. Previously reported histone degradation can be related to DNA repair [62, 63].

It was further indicated that the expression of HSP70, heat shock protein, increases during anastasis and plays a vital role in reducing DNA damage [64]. This protein prevents DNA degradation through interaction with apoptosis-inducing factor (AIF) and the mitochondrial protein, endonuclease G (EndoG) (promotes caspase-independent apoptosis via DNA degradation) elements [65]. In addition, the expression of several factors involved in cell cycle arrests, such as B-cell translocation gene 1 (BTG1), cyclin-dependent kinase inhibitor 1A (CDKN1a), and tumor protein p53-inducible nuclear protein 1 (TP53INP1), increased during anastasis, which can be associated with facilitating DNA repair [66, 67].

Although various DNA repair systems are activated during anastasis, the cell cannot repair all DNA damage. Hence, micronuclei formation, DNA breaks, and chromosomal instability are observed in most anastatic cells [68]. Accumulation of DNA breakage can cause the transformation of normal cells to cancer cells and tumor cells to become more malignant [69].

### Recovery through apoptotic bodies formation and phosphatidylserine

Cells fragmentation into apoptotic bodies followed by phagocytosis is the final stage of apoptosis. Even in this case, anastasis can occur after cell fragmentation [70]. In a recent study, Tang et al. demonstrated that HeLa cervical cancer cells could fuse and form seemingly normal morphology after brief incubation with staurosporine apoptotic bodies [67]. Another study by Raj et al. reported that fragments of the H446 lung cancer cells could fuse and form normal morphology followed by induction of apoptosis by ethanol [71]. However, anastatic cells commonly have many chromosomal abnormalities and micronuclei, indicating incorrect fuse of apoptotic bodies with damaged chromosomes to form a mononuclear cell. This process can cause to increase the rate of aneuploidy in cancer cells [8, 66, 71].

The exact underlying mechanisms of anastasis and recovery of normal cell function and morphology through apoptotic bodies fusion remain unknown. Previous studies reported that the presence of phosphatidylserine is an essential factor for different types of cell fusion, which shows that fragmented could use this strategy for fusion and repair during anastasis [8]. It can be said that cell fusion and recovery can occur through similar mechanisms in apoptotic bodies with externalized phosphatidylserine. The phosphatidylserine is an essential component of the inner plasma membrane. During apoptosis is transferred to the outer surface of the plasma membrane and represents a signal for facilitating the phagocytes' function [72, 73]. However, anastasis can occur even with transferred phosphatidylserine to the surface of the membrane. Evidence suggested that B lymphoma cells and mouse mammary carcinoma cells could recover after surface exposure to phosphatidylserine [74, 75]. In addition, anastasis has been observed after phosphatidylserine exposure in mouse cardiac muscle HL1 cell line and neonatal rat primary cardiomyocytes after induction of cell death through ethanol treatment. The anastatic cells are likely to remove phosphatidylserine signals and cause to escape phagocytosis [8, 66]. A study by Kenis et al. reported that phosphatidylserine transferred to the inner plasma membrane in recovered cardiomyocytes cells after ischaemic injury [76]. Although exposure to PS in the outer leaflet of the plasma membrane is a crucial feature of apoptosis, caspase-independent PS exposure has been shown to occur in primary T cells during apoptosis induced by stimuli that do not trigger death receptors [77]. Therfore, further studies are required to recognize molecular pathways of phosphatidylserine internalization.

## Drug resistance and anastasis

Drug resistance in cancer therapy is one of the most critical problems in cancer chemotherapeutic methods [78]. Several proteins are involved in DNA repair, proapoptotic, and anti-apoptotic mechanisms (NFκB, PARP-1, MDM2, c-FLIP, survivin, BCL-2, MCL-1, and BCL-XL) are associated with drug resistance and tumor recurrence. These factors promoted drug resistance in cancer cells by increasing aggressive characteristics of the tumor (immune evasion, cellular dormancy, cancer stemness, metastasis, and migration) [79, 80].

Anastasis is included in associated processes in both pro-metastatic and pro-survival and is an essential factor in drug resistance in cancer therapy. Evidence suggested an association between anastasis and drug resistance, as well as the involvement of anastasis-related genes and pathways, facilitate drug resistance to chemotherapeutic agents [81]. In this regard, a previous study by Seervi et al. reported an increased expression of exportin 1 (XPO1) gene in anastatic breast and cervical cancer cells recovered after transition treatment with etoposide and paclitaxel as anticancer compounds (XPO1 gene encodes a nuclear export protein which is involved in the promotion of acquired drug resistance). They demonstrated that downregulation of XPO1 significantly decreases apoptotic cells recovery and abrogate oncogenic transformation in anastatic cancer cells. In addition, upregulation of more than forty proteins (involved in Ras, redox, and nuclear export/import signaling pathways) in anastatic breast and cervical cancer cells indicates common molecular mechanisms in cells. They suggested that inhibition of anastasis through modification of nuclear export pathway may be a potential therapeutic approach for overcoming metastasis, drug-resistance, and other problems during cancer therapy [82]. Another study by Fresquet et al. demonstrated that periodic treatment of murine lymphoma cells (LyBcl2-9 and LyBcl2-6) by venetoclax (BH-3-mimetic ABT-199) leads to acquired resistance. They identified two missense mutations in the Bcl2-BH3 domain in Bcl2-expressing resistant lymphoma cells, which cause to suppression of mitochondrial apoptosis through prevention of ABT-199 to the BH3 domain. In addition, they found a missense mutation (G179E) in proapoptotic BAX (at C-terminal transmembrane domain) in resistant human lymphoma cells, which block anchoring of BAX to mitochondria and inhibit ABT-199 induced apoptosis. Importantly, this mutation in BAX promotes partial resistance to antineoplastic agents [83].

Since cancer chemotherapy is currently conducted on the principle of periodic drug administration, chemotherapeutic methods based on occasional treatment may increase drug resistance through anastasis-related mechanisms [84]. Due to the probability of tumor recurrence after-acquired drug resistance following periodic anticancer chemotherapy, further studies are required to identify anastasis's role in cancer drug resistance.

## Tumor progression factors and anastasis

Cancer cell survival is dependent on several processes such as upregulation of anti-apoptotic genes, dysregulation of the cell cycle, transforming growth factor-beta (TGF-β) modulation, angiogenesis, migration, and defects in DNA repair [53], which are highly activated during early anastasis [85]; whereas, activated factors in late anastasis includes biogenesis of ribosome, arrangements of the actin cytoskeleton, pathways of focal adhesion, and processing of proteins [86]. In addition, activation of activator protein 1 (AP1) transcription factor oncogenes such as c-Fos and c-Jun, which can act as pro-apoptotic or pro-survival factors dependent on the cellular context, are essential in anastasis [87]. Therefore, after apoptosis induction through TRAILR2, a receptor-dependent pathway, recovered cells can form a malignant tumor with metastasis potential [88].

Induction of epithelial-mesenchymal transition (EMT) by anti-apoptotic factors in cell survival is an essential feature of anastasis [86]. Moreover, upregulation of the zinc finger protein SNAI1 (SNAIL1) transcription factor is necessary for anastasis of cancer cells, which stimulates EMT through inhibition of E-cadherin synthesis [89]. Several other signaling pathways, such as Wnt, TGFβ, and Ras/Mitogen-activated protein kinase (Ras/MAPK), are activated in the initiation of anastasis, which is associated with cell survival and EMT induction [90]. In addition, the evidence demonstrated a high expression in matrix metalloproteinase (MMP-9, MMP-10, and MMP-13) in the late anastasis [86]. Transformation of tumor cells to cancer stem cells is facilitated by anastasis, which can cause more drug-resistant and neoplasms recurrence. A previous study by Xu et al. reported that expression of CD44, stemness marker, is increased in anastatic breast cancer cells [91]. Additionally, the expression of vascular growth factor-A (VEGFA) and angiopoietin-like protein-4 (ANGPL-4) is increased during anastasis in cancer cells [10].

## Conclusion

**Fig. 1:**
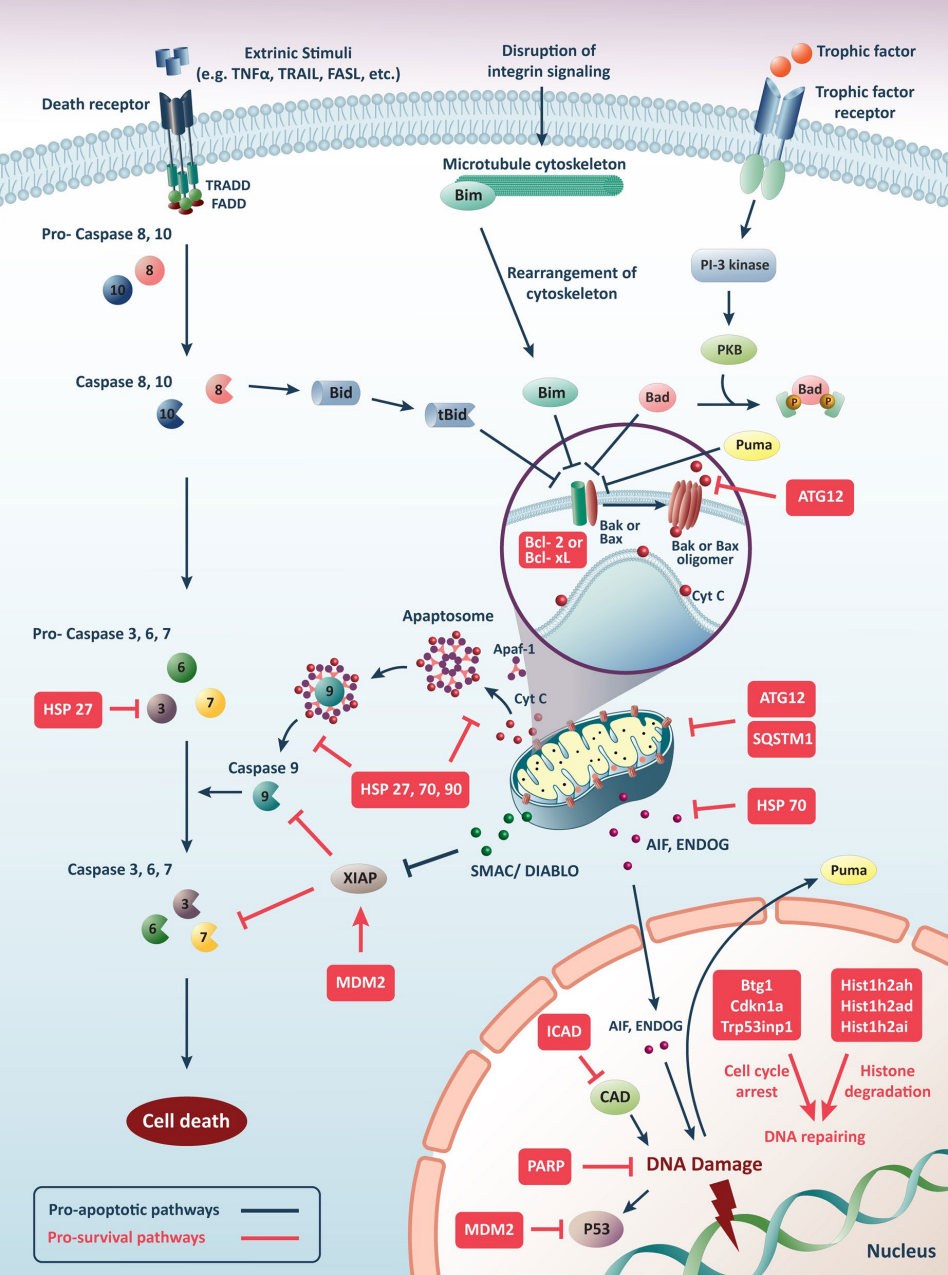
Overview of pro-apoptotic and pro-survival signaling pathways and a proposed mechanism of anastasis or cell recovery. This figure shows that extrinsic stimuli such as TNF alpha, TRAIL, FASL, and so on can initiate apoptotic cascaded by binding to death receptors and activating caspase 8 and 10, which in turn activate caspases 3, 6 and 7, leading to cell death. In this route, cells can rescue themselves by induction of HSP27 to suppress effector caspases (3, 6 and 7) and inhibit apoptosis. Activated caspases 8 and 10 produced in this way can activate BID and tBID, leading to apoptosis by suppressing BAX and BAK. However, if cells survive, they can inhibit pro-apoptotic BAX and BAK factors by pro-survival factors reinforcement. On the other hand, disruption of cell membrane integrity causes BIM and BAD activation and rearrangement of cytoskeleton, leading to apoptosis by suppressing BAX and BAK. However, when cells sense a survival signal, they can stop pro-apoptotic BAX and BAK factors that BIM and BAD induce through reactivating pro-survival factors such as BCL-2 and BCL-XL. Trophic factors can activate PI3K and PKB, leading to BAD and PUMA activation that causes BCL-2 and BCL-XL hindering. However, this route can be reversed through ATG12 activation and suppressing BAX and BAK if cells need survival. Moreover, Because of various inducers, Cytochrome c is released into the cytoplasm and activates Apaf-1, causing the ring-like apoptosome to form and activate caspase 9, which activates the effector caspases (3, 6 and 7). However, suppose cells sense a signal to hinder apoptosis. In that case, they can perform it by various molecules, such as SQSTM1 and ATG12, which suppresses cytochrome release, HSP 27, 70, and 90 that inhibit the formation of apoptosome and caspase 9 activations, and XIAP that inhibit activation of caspase 9 and effector caspases. At the same time, XIAP can be inhibited by Smac/DIABLO to initiate apoptosis. When the permeability of mitochondria is lost due to apoptosis-inducer factors, AIF and EndoG are released and trigger apoptosis through activating signaling pathways inside the nucleus, while they can be inhibited HSP70 if cells need to be rescued; otherwise, they cause DNA damage and cell death. However, this route can be inhibited by molecules inside the nucleus such as ICAD, PARP-1, MDM2, BTG1, CDKN1A, P53INP1, and Hist1h. TNFα: Tumor necrosis factor; TRAIL: TNF-related apoptosis-inducing ligand; FASL: Fas ligand; BID: BH3-interacting domain death agonist; tBID: truncated BID; BAX: BCL2 Associated X, Apoptosis Regulator; BAK: BCL2 Antagonist/Killer 1; BIM: Bcl-2-like protein 11; BAD: BCL2 associated agonist of cell death; PI3K: Phosphoinositide 3-kinase; PKB: Protein kinase; PUMA: p53 upregulated modulator of apoptosis APAF: Apoptotic protease activating factor-1; SQSTM1: Sequestosome 1: ATG: Autophagy protein; HSP: Heat shock protein; XIAP: X-linked inhibitor of apoptosis protein; Smac: Second mitochondria-derived activator of caspase; DIABLO: Inhibitor of apoptosis protein (IAP)-binding protein with low pI (DIABLO); AIF: Apoptosis-inducing factor; EndoG: Endonuclease G; CAD: Caspase-activated DNase; ICAD: Inhibitor of caspase-activated DNase; PARP-1: poly(ADP-ribose) polymerase-1; MDM2: Mouse double minute 2 homolog; BTG1: BTG Anti-Proliferation Factor 1; CDKN1A: Cyclin-Dependent Kinase Inhibitor 1A; P53INP1: Tumor Protein P53 Inducible Nuclear Protein; Hist1h: Histone H2A type 1-H

Identification of molecular pathways of apoptosis followed by biochemical mechanisms can be crucial in cancer therapies by inducing apoptosis in malignant cells. Anastasis is a novel concept in cancer biology and the treatment of malignancies. Cell death is a reversible event, and cells can revive after apoptosis. The anastatic cells are employed usual pro-survival and pro-metastatic factors to inhibit the progression of apoptosis. The cell death/survival balancing is essential for normal homeostasis and development and can prevent diseases and cancer. The unbalanced function of anti-apoptotic and pro-apoptotic factors can cause dysregulated cell death or survival. All the text-mentioned routes have been demonstrated in Fig. 1. Anastasis can increase drug resistance and metastasis of various cancer cells, a significant cause of cancer mortality. However, our knowledge of mechanisms of anastasis is limited, and further studies are essential to the development of therapeutic strategies based on anastasis.

## Data Availability

Not applicable.

## Abbreviations

MOMP: Mitochondrial outer membrane permeabilization
APAF1: Apoptotic protease activating factor 1
Bcl-2: B-cell lymphoma 2
Bcl-XL: B-cell lymphoma-extra-large
Mcl-1: Myeloid cell leukemia-1
FASLG: FAS-ligand
TRAIL: Tumor necrosis factor–related apoptosis-inducing ligand
TNFα: Tumor necrosis factor alpha
BH3: Bcl-2 Homology 3
BID: BH3 interacting-domain death agonist
ATG12: Autophagy related 12
SQSTM1: Sequestosome 1
DNAJB1: DnaJ homolog subfamily B member I
GAPDH: Glyceraldehyde-3-phosphate dehydrogenase
HSP: Heat shock protein
MDM2: Mouse double minute 2 homolog
XIAP: X-linked inhibitor of apoptosis
CAD: Caspase-activated DNase
ICAD: Inhibitor of CAD
PARP: Poly ADP-ribose polymerase
Hist1h2a: Histone H2A
EndoG: Mitochondrial protein, endonuclease G
AIF: Apoptosis-inducing factor
BTG1: B-cell translocation gene 1
CDKN1a: Cyclin-dependent kinase inhibitor 1A
TP53INP1: Tumor protein p53-inducible nuclear protein 1
NF-κB: Nuclear factor kappa-light-chain
c-FLIP: Cellular FLICE (FADD-like IL-1β-converting enzyme)-inhibitory protein
XPO1: Exportin 1
TGF-β: Transforming growth factor beta
AP1: Activator protein 1
EMT: Epithelial-mesenchymal transition
SNAIL1: Zinc finger protein SNAI1
Wnt: Wingless-related integration site
Ras/MAPK: Ras/mitogen-activated protein kinase
MMP: Matrix metalloproteinase
VEGFA: Vascular growth factor-A
ANGPL-4: Angiopoietin-like protein-4
CD44: Cluster of differentiation 44
