# Correction: Anastasis: cell recovery mechanisms and potential role in cancer

**DOI:** 10.1186/s12964-022-00914-3

**Published:** 2022-06-16

**Authors:** Rebar N. Mohammed, Mohsen Khosravi, Heshu Sulaiman Rahman, Ali Adili, Navid Kamali, Pavel Petrovich Soloshenkov, Lakshmi Thangavelu, Hossein Saeedi, Navid Shomali, Rozita Tamjidifar, Alireza Isazadeh, Ramin Aslaminabad, Morteza Akbari

**Affiliations:** 1 Medical Laboratory Analysis Department, College of Health Sciences, Cihan University of Sulaimaniya, Kurdistan Region, Sulaimaniyah, Iraq; 2 College of Veterinary Medicine, University of Sulaimani, Sulaimaniyah, Iraq; 3 Department of Psychiatry and Clinical Psychology, Zahedan University of Medical Sciences, Zahedan, Iran; 4 Immunology Research Center, Tabriz University of Medical Sciences, Tabriz, Iran; 5 Department of Physiology, College of Medicine, University of Sulaimani, Sulaimaniyah, Iraq; 6 Department of Medical Laboratory Sciences, Komar University of Science and Technology, Sarchinar District, Sulaimaniyah, Iraq; 7 Department of Oncology, Tabriz University of Medical Sciences, Tabriz, Iran; 8 I. M. Sechenov First Moscow State Medical University, Ministry of Health of the Russian Federation (Sechenov University), Moscow, Russia; 9 Department of Pharmacology, Saveetha Dental College, Saveetha Institute of Medical and Technical Science, Saveetha University, Chennai, India

## Correction: Cell Communication and Signaling (2022) 20:1–9 10.1186/s12964-022-00880-w

Following publication of the original article [1], the authors identified an error in the following affiliation:

1. Medical Laboratory Analysis Department, College of Health Sciences, Cihan University of Sulaimaniya, Kurdistan Region, Iraq.

Cihan University was misspelt as Cihlan University.
